# Mediation analysis of activities of daily living and kinesiophobia in association between cardiac function and health status of patients with chronic heart failure

**DOI:** 10.1002/clc.24147

**Published:** 2023-09-14

**Authors:** Tang Yifan, Huang Yanling, Wang Weiyun, Hu Xiaolin, Gu Zejuan, Wang Rong, Gao Chunhong

**Affiliations:** ^1^ Department of Geriatric Cardiology The First Affiliated Hospital with Nanjing Medical University Nanjing Jiangsu Province PR China; ^2^ Department of Nephrology The First Affiliated Hospital, Sun Yat‐sen University Guangdong PR China; ^3^ Department of Cardiovascular Surgery The First Affiliated Hospital with Nanjing Medical University Nanjing Jiangsu PR China; ^4^ Secretariat of Party Committee, The First Affiliated Hospital with Nanjing Medical University Nanjing Jiangsu PR China; ^5^ Nursing Department The First Affiliated Hospital with Nanjing Medical University Nanjing Jiangsu PR China

**Keywords:** activities of daily living, cardiac function, chronic heart failure, health status, kinesiophobia, mediational effect

## Abstract

**Aims:**

To explore the mediational effect of activities of daily living (ADL) and kinesiophobia on the cardiac function and health status of patients with chronic heart failure (CHF).

**Methods:**

From October 2021 to January 2022, a total of 244 CHF patients treated in the Department of Cardiology of general hospitals were recruited by the convenience sampling method. They were investigated with the Tampa Scale for Kinesiophobia Heart (TSK‐SV Heart), the Barthel index for assessing ADL, and the EuroQol five‐dimensional questionnaire (EQ‐5D) for assessing the health status.

**Results:**

The cardiac function and kinesiophobia of CHF patients were both negatively correlated with their health status (*r* = −.390 and −0.410, respectively, both *p* < .01). Besides, the ADL of CHF patients was positively correlated with the health status (*r* = .320, *p* < .01). The cardiac function of CHF patients was negatively correlated with the ADL (*r* = −.412, *p* < .01), but positively correlated with kinesiophobia (*r* = .180, *p* < .01). The mediation proportion of ADL plus kinesiophobia between the cardiac function and health status of CHF patients was 43.48%. Both ADL and kinesiophobia partially mediated the effect of cardiac function on health status in CHF patients, but their mediational effects showed no significant difference (*p* = .777).

**Conclusion:**

Both ADL and kinesiophobia exert obvious mediational effects between cardiac function and health status in CHF patients. Individualized cardiac rehabilitation (CR) programs based on the cardiac function, ADL and kinesiophobia of CHF patients may contribute to reduce the medical burden and improve the well‐being of affected people.

## Graphical abstract

1. Only 2.5% of chronic heart failure(CHF) patients were engaged in Cardiac rehabilitation(CR) programs and 77.9% of them participated in daily activities/exercise, the top three of which were walking (73.8%), housework (27.9%) and jogging (21.3%). Kinesiophobia was detected in 170/244 (69.5%) CHF patients who had limited or low‐intensity daily activities/exercises. Both activity of Daily Living (ADL) and kinesiophobia partially mediated the effect of cardiac function on health status in CHF patients, the mediational effects are equal. Kinesiophobia should be relieved and ADL enhanced to improve the health status and prolong the life expectancy of CHF patients.

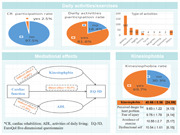

## 1 INTRODUCTION

Heart failure (HF), resembling a malignant tumor of heart disease, has become a global health issue, due to its high morbidity, mortality, readmission rate and medical cost.^1^, ^2^ HF patients suffer from poor activities of daily living (ADL) and quality of life due to severe dyspnea, fatigue, insomnia and anxiety.^1^, ^2^, ^3^ Cardiac rehabilitation (CR), as a new research focus in cardiology, has been validated as a stage 1 intervention for lowering the risk of cardiovascular diseases.^4^, ^5^, ^6^ Exercise rehabilitation, as the core strategy of CR, can effectively improve the physical health and quality of life, and reduce the mortality.^7^, ^8^ Kinesiophobia is defined as an excessive fear of possible injury in physical movements and activities. It can increase the incidences of somatic symptoms and movement disorders, which eventually threatens human health.^9^, ^10^ Previous studies have demonstrated that the health status of HF patients is affected by cardiac function, ADL and kinesiophobia, and a pairwise relationship exists.^10^, ^11^, ^12^, ^13^ However, the correlations among the health status, cardiac function, ADL and kinesiophobia of HF patients remain unclear. In the present study, we aimed to explore the mediational effect of ADL plus kinesiophobia between cardiac function and health status in chronic HF (CHF) patients, thus providing theoretical references for improving the health of affected people.

## 2 METHODS

### 2.1 Subjects

A consecutive sample of 244 HF patients were recruited from the Department of Cardiology of the First Affiliated Hospital with Nanjing Medical University from October 2021 to January 2022 by convenience sampling method. Inclusion criteria: (1) CHF diagnosed based on the *Chinese Guidelines for Diagnosis and Treatment of Heart Failure 2018*; (2) Age ≥ 24 years; (3) CHF course ≥ 5 months; (4) Class Ⅰ−Ⅳ cardiac function; (5) Written informed consent obtained. Exclusion criteria: (1) Subjects with unconsciousness or cognitive impairment; (2) Subjects with malignant tumors, severe liver or renal failure. This study was approved by the Ethic Committee of the First Affiliated Hospital with Nanjing Medical University (2019‐SR‐474).

### 2.2 Questionnaires

Pretrained investigators were responsible for explaining the aim and clinical significance of this study using the unified instructions. The subjects were asked to fill in questionnaires by themselves after obtaining the written informed consent. For those who were unable to write or read by themselves, investigators read the items one by one, and recorded their answers. All completed questionnaires were collected within 48 h before discharge, and checked on the spot. All missed or incorrectly filled items were revised by reinquiring subjects.

#### 2.2.1 Questionnaire for baseline characteristics

The self‐designed questionnaire for 14 baseline characteristics of subjects included the sex, age, marriage, educational level, occupation, current work situation, current per capita monthly household income (yuan), medical insurance, smoking history, drinking history, comorbidities at admission, course of HF, cardiac function classification, and primary disease.

#### 2.2.2 Questionnaire for exercise status and type

The self‐designed questionnaire for exercise status and type included the involvement of CR programs, daily exercises/activities, the frequency, time and type of exercises/activities, and fitness centers near your home.

#### 2.2.3 Barthel index

The 10‐item Barthel index was calculated for assessing ADL, with a total score of 100 points. A higher Barthel index indicated better ADL.

#### 2.2.4 Tampa scale for kinesiophobia heart (TSK‐SV Heart)

The Chinese version of TSK‐SV Heart developed by Lei et al.^14^ in 2019 was a 17‐item scale with four factors, including perceived danger for heart problem, fear of injury, avoidance of exercise and dysfunctional self. Using a 4‐point Likert scale, the subjects with a total score ≥ 37 scores were diagnosed as kinesiophobia. Cronbach's *α* for the TSK‐SV Heart was .859.

#### 2.2.5 EuroQol five‐dimensional questionnaire (EQ‐5D)

The 6‐item EQ‐5D was used to assess health status in five dimensions, including the mobility, self‐care, usual activities, pain/discomfort and anxiety/depression.^15^ Cronbach's *α* for the EQ‐5D was 0.761. The EQ‐5D score was transformed using the time trade‐off model.^16^

### 2.3 Statistical analysis

Statistical analysis was performed using SPSS 25.0. Enumeration data were expressed as the frequency and constituent ratio, and measurement data as mean ± standard deviation. The correlation between two variables was identified by the Spearman's rank correlation. Analysis of variance single factor analysis of variance was used. The structural equation modeling and path analysis were performed using IMB SPSS Amos 26.0. The mediation analysis with two mediators was performed by the bootstrap test at *α* = .05. *p* < .05 was considered as statistically significant.

## 3 RESULTS

### 3.1 Baseline characteristics of CHF patients and univariate analyses of kinesiophobia

A total of 244 eligible CHF patients with a mean age of 64.45 ± 11.076 (30–93) years and a mean number of 3.05 ± 1.796 (1, 10) comorbidities were recruited, involving 138 (56.6%) men and 106 (43.4%) women. Their baseline characteristics were listed in Table 1. Patients with HF who had a minimum TSK‐SV Heart score of 37 points were defined as Kinesiophobia group. There was an overall statistically significant result of occupation, educational level, as well as current per capita monthly household income on Kinesiophobia score.

**Table 1:** Baseline characteristics of the subjects and single factor analysis of Kinesiophobia among patients with CHF (*n* = 244).

Item	Kinesiophobia	Without Kinesiophobia	Test statistic	*p* Value
	Case number (*n*, %)	Case number (*n*, %)		
Age			0.571	.569
30−45	5 (2.9%)	6 (8.1%)		
46−60	61 (35.9%)	25 (33.8%)		
61−75	85 (50.0%)	33 (44.6%)		
76−90	19 (11.2%)	10 (13.5%)		
Male	98 (57.6%)	40 (54.1%)	−0.516	.607
Marriage			−0.45	.667
Unmarried	9 (5.3%)	1 (1.4%)		
Married	161 (94.7%)	70 (94.6%)		
Divorced	3 (1.8%)	1 (1.4%)		
Widowed	5 (2.9%)	3 (4.1%)		
Educational level			−4.242	0
Primary school and below	59 (34.7%)	11 (14.9%)		
Junior high school	57 (33.5%)	19 (25.7%)		
High school or secondary technical school	27 (15.9%)	16 (21.6%)		
Junior collage	11 (6.5%)	17 (23%)		
Bachelor degree or above	16 (9.4%)	11 (14.9%)		
Occupation			−2.623	.007
Workers or peasants	114 (67.1%)	28 (37.8%)		
Civil servants or cadres	7 (4.1%)	12 (16.2%)		
Scientific and technical workers	26 (15.3%)	22 (29.7%)		
Freelancers or servicers	14 (8.2%)	11 (14.9%)		
Others	10 (4.1%%)			
Current work situation			0.664	.488
Retired	84 (49.4%)	39 (52.7%)		
Sick leave	72 (42.4%)	29 (39.2%)		
On the job	0 (0.0%)	3 (4.1%)		
Others	14 (8.2%)	3 (4.1%)		
Current per capita monthly household income (yuan)			−4.728	0
1000–3000	23 (13.5%)	1 (1.4%)		
3001–5000	82 (48.2%)	23 (31.1%)		
≥5001	65 (38.2%)	50 (67.6%)		
Medical insurance			−0.96	.315
Self payment	36 (21.2%)	11 (14.9%)		
Rural cooperative medical system	1 (0.6%)	1 (1.4%)		
Medical insurance	121 (71.2%)	57 (77%)		
Cadre health care	12 (7.1%)	5 (6.8%)		
Smoking history	26 (15.3%)	9 (12.2%)	−0.808	.42
Drinking history	29 (17.1%)	17 (23%)	0.854	.394
Course of disease (years)			0.495	.621
<1	39 (22.9%)	20 (27%)		
1−5	76 (44.7%)	37 (50%)		
5.1−10	34 (20.0%)	7 (9.5%)		
>10	21 (12.4%)	10 (13.5%)		
Cardiac function classification			1.361	.173
Class Ⅰ	9 (5.3%)	7 (9.5%)		
Class Ⅱ	102 (60.0%)	46 (62.2%)		
Class Ⅲ	48 (28.2%)	18 (24.3%)		
Class Ⅳ	11 (6.5%)	3 (4.1%)		
Primary disease			0.956	.334
Hypertension	3 (1.8%)	4 (5.4%)		
Coronary heart disease	70 (41.2%)	31 (41.9%)		
Arrhythmia	48 (28.2%)	17 (23%)		
Valvular disease	25 (14.7%)	17 (23%)		
Cardiomyopathy	24 (14.1%)	5 (6.8%)		

Abbreviation: CHF, chronic heart failure.

### 3.2 Daily activities/exercises of CHF patients

Only 6 (2.5%) of CHF patients used to or were now participating in CR programs. A total of 190 (77.9%) CHF patients could perform ADL. The frequency, time and type of daily activities/exercises are listed in Table 2.

**Table 2:** Exercise status and type of CHF patients (*n* = 190).

Item	Case number (*n*, %)
Frequency of exercises/activities per week	
1	4 (1.6%)
2−3	32 (13.1%)
4−5	119 (48.8%)
>5	35 (14.3%)
Time of exercises/activities (h)	
<0.5	26
0.5−1	121
1−2	41
>2	2 (0.8%)
Type of exercises/activities	
Walking	180 (73.8%)
Dancing	17 (7.0%)
Tai chi	4 (1.6%)
Ball games	3 (1.2%)
Swimming	4 (1.6%)
Gymnastics	2 (0.8%)
Housework	68 (27.9%)
Jogging	52 (21.3%)
Others	44 (18.0%)

Abbreviation: CHF, chronic heart failure.

### 3.3 Kinesiophobia, ADL and health status of CHF patients

Kinesiophobia was detected in 170 (69.5%) of CHF patients who had a minimum TSK‐SV Heart score of 37 points. There were 202 (82.2%) with a minimum Barthel index of 60‐points, suggesting their good ADL (Table 3).

**Table 3:** Kinesiophobia, ADL and health status of CHF patients (*n* = 244).

Item	Scores (points)
Kinesiophobia	
Perceived danger for heart problem	9.83 ± 1.22 (4.00−13.00)
Fear of injury	9.75 ± 1.78 (4.00−14.00)
Avoidance of exercise	10.55 ± 2.70 (5.00−17.00)
Dysfunctional self	10.54 ± 1.61 (6.00−15.00)
Total	40.66 ± 5.56 (24.00−59.00)
ADL	
Grooming	4.16 ± 1.54 (0−5.00)
Bathing	3.42 ± 2.01 (0−5.00)
Feeding	8.34 ± 2.44 (0−10.00)
Toilet use	8.13 ± 2.74 (0−10.00)
Dressing	8.18 ± 2.68 (0−10.00)
Bowels	9.89 ± 0.63 (5−10.00)
Bladder	9.74 ± 1.24 (0−10.00)
Stairs	6.66 ± 3.68 (0−10.00)
Transfer	11.94 ± 4.36 (0−15.00)
Mobility	11.66 ± 4.67 (0−15.00)
Total	82.24 ± 21.36 (20.00−100.00)
Health status	
Mobility	0.0228 ± 0.0379 (0−0.2668)
Self‐care	0.0306 ± 0.0786 (0−0.4441)
Usual activities	0.0201 ± 0.0198 (0−0.0538)
Pain/discomfort	0.0216 ± 0.0112 (0−0.0274)
Anxiety/depression	0.0146 ± 0.0177 (0−0.0359)
Quality of life	0.1096 ± 0.1197 (0−0.5444)
Self‐feeling health status	75.22 ± 12.90 (30.00−100.00)
Total	0.8904 ± 0.1197 (0.4556−1.00)

Abbreviations: ADL, activities of daily living; CHF, chronic heart failure.

### 3.4 Correlation among the cardiac function, health status, ADL and kinesiophobia of CHF patients

It is shown that the cardiac function of CHF patients was negatively correlated with the health status and ADL (*p* < .01), but positively correlated with the kinesiophobia (*p* < .01). In addition, ADL of CHF patients was positively correlated with the health status (*p* < .01), but negatively correlated with the kinesiophobia (*p* < .01). A negative correlation was identified between kinesiophobia and health status in CHF patients (*p* < .01) (Table 4).

**Table 4:** Correlations among the cardiac function, health status, ADL and kinesiophobia of CHF patients (*n* = 244).

	Cardiac function (*r*)	Health status (*r*)	ADL (*r*)	Kinesiophobia (*r*)
Cardiac function	‐	−0.390	0.180	−0.412
*p* Value	‐	.000	.005	.000
Health status	−0.390	‐	−0.410	0.320
*p* Value	.000	‐	.000	.000
ADL	−0.412	0.320	‐	−0.187
*p* Value	.000	.000	‐	.003
Kinesiophobia	0.180	−0.410	−0.187	‐
*p* Value	.005	.000	.003	‐

Abbreviations: ADL, activities of daily living; CHF, chronic heart failure.

### 3.5 Mediational effects of kinesiophobia and ADL between cardiac function and health status of CHF patients

**Figure 1:**
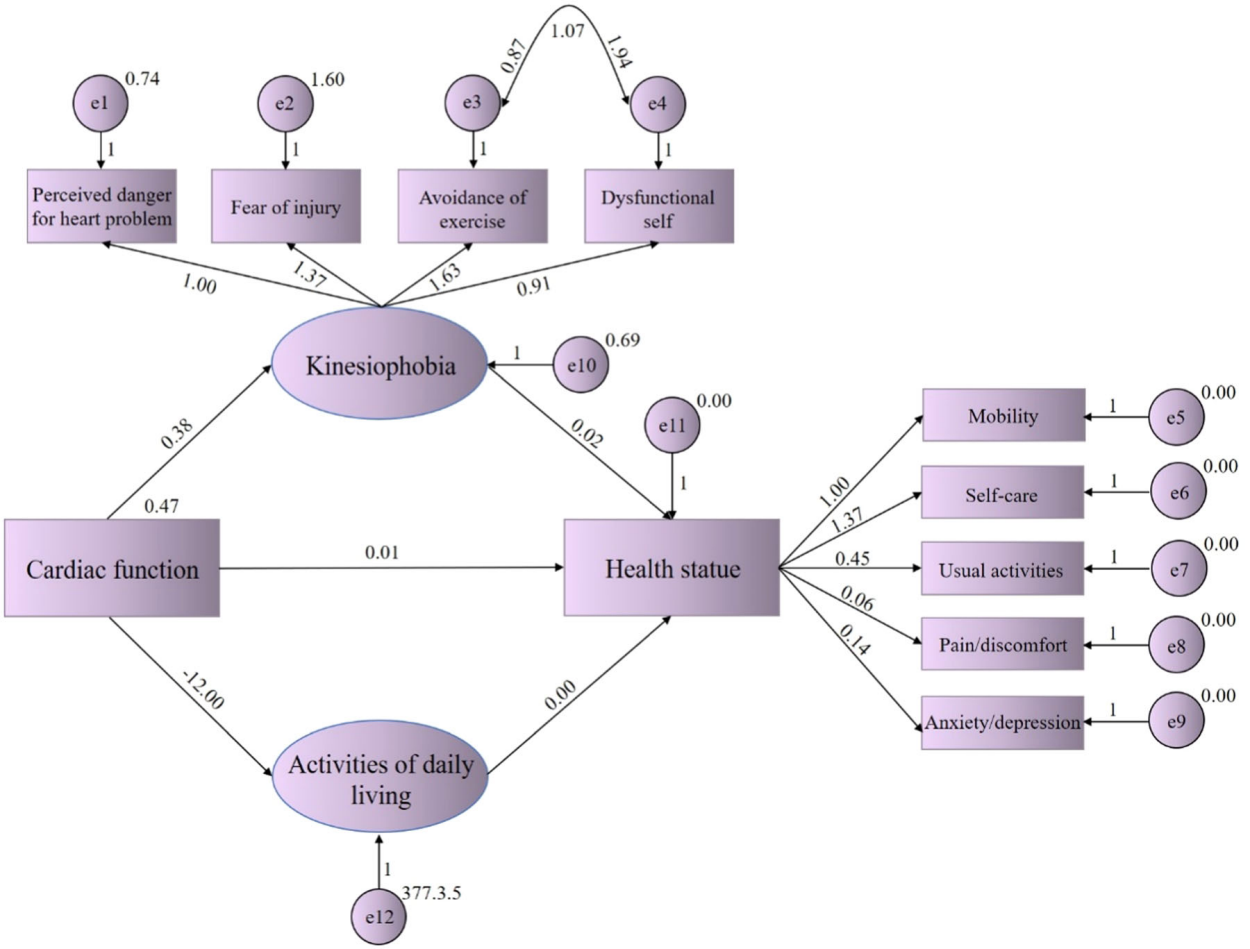
A structural equation model of activities of daily living and kinesiophobia between cardiac function and health status of patients with chronic heart failure.

We created a structural equation model, in which the cardiac function was the independent variable, kinesiophobia and ADL were mediation factors, and health status was the dependent variable (Figure 1). After adjustment, *χ* ^2^/*df*, goodness‐of‐fit index (GFI), adjusted GFI (AGFI), comparative fit index, Tucker‐Lewis index and the root mean square error or approximation of the model were 1.614, 0.954, 0.924, 0.951, 0.933, and 0.053, suggesting the good fitness of the structural equation model. Furthermore, the significance of mediational effects was tested by bootstrapping in IMB SPSS Amos 26.0 with the sample size of 5000. It is shown that the 95% confidence interval (CI) of bootstrap was 0.036−0.532, in which 0 was not included, suggesting the statistical significance of mediational effects of kinesiophobia and ADL between cardiac function and health status of CHF patients. In the mediation analysis, the total effect c and the direct effect c΄ were 0.023 (*p* < .01) and 0.013 (*p* = .001), respectively. The indirect effect (ab) was calculated as ab = a1 × b1 + a2 × b2 = 0.006 + 0.004 = 0.01. Therefore, the mediation proportion of ADL plus kinesiophobia in the correlation between cardiac function and health status, which was calculated as the ratio of the indirect effect to the total effect, was 43.48%. The difference between the mediational effects of two mediators (ADL and kinesiophobia) was 0.001 (the bootstrap standard error = 0.333; 95% CI, −0.004 to 0.007; *p* = .777), suggesting that both ADL and kinesiophobia partially mediated the effect of cardiac function on health status of CHF patients, but their mediational effects had no significant difference (Table 5).

**Table 5:** Mediational effects of kinesiophobia and ADL between cardiac function and health status of CHF patients (*n* = 244).

Path	Mediational effect	Bootstrap SD	Bootstrap 95% CI	*p* Value	Mediation proportion
Indirect effect 1 (cardiac function→kinesiophobia→health status)	0.006	0.333	0.002−0.013	.000	26.09%
Indirect effect 2 (cardiac function→ADL→health status)	0.004	0.250	0.002−0.007	.001	17.39%
Indirect effect (cardiac function→kinesiophobia + ADL→health status)	0.010	0.300	0.005−0.016	.000	43.48%
Direct effect (cardiac function→health status)	0.013	0.308	0.006−0.022	.001	56.52%
Total effect	0.023	0.174	0.015−0.032	.000	100.00%

Abbreviations: ADL, activities of daily living; CHF, chronic heart failure; CI, confidence interval; SD, standard deviation.

## 4 DISCUSSION

### 4.1 Single factor analysis of kinesiophobia in patients with HF

The findings of this study indicate a significant association between kinesiophobia score and occupation, educational level, as well as current per capita monthly household income. Specifically, individuals in occupations such as workers and farmers, those with lower levels of education, and those with lower current per capita monthly household income were found to be more vulnerable to kinesiophobia among patients with HF. These findings are consistent with prior research conducted by Zhang et al.^17^ The primary factors contributing to this phenomenon are as follows: Individuals exhibiting these clinical characteristics often find themselves in a socially disadvantaged and insecure position, burdened by significant economic and psychological challenges. Consequently, they face increased difficulty in managing potential exercise‐related adverse events. Further investigation is necessary to devise recruitment strategies specifically tailored to vulnerable subgroups. Additionally, during the initial stages of the disease, efforts should be made to enhance patients' activity through diverse approaches to health education. This entails elucidating the benefits of CR exercises and the detrimental effects of a sedentary lifestyle, thereby instilling a belief in the importance of active physical activity among affected individuals. Simultaneously, government must ensure the provision of sustainable health care services to alleviate concerns surrounding CR and foster increased participation rates.

Our results indicate that there is no statistically significant relationship between gender or age variables and kinesiophobia scores. It can be seen that the same result in individuals with coronary heart disease, heart transplant or burn injury.^18^, ^19^, ^20^ At present, kinesiophobia severity dependence on age or gender in patients with cardiovascular diseases still remains controversial. Nonetheless, previous studies have indicated notable gender and age disparities in CR referral, registration, and completion rates.^21^, ^22^ It is likely associated with transportation problems, family responsibilities, having multiple medical issues, and the perception of exercise as tiring or painful. These factors collectively contribute to the reluctance of individuals to enroll in CR programs. This finding also suggests that the kinesiophobia may be intricate in patients with cardiovascular disease, particularly in relation to psychological, social and environment‐related factors. Therefore, it has been advisable to incorporate supplementary measures, such as the Social Support Scale and the Positive Psychology Scale, to enhance the comprehensiveness of the investigation.

### 4.2 Exercises, ADL and kinesiophobia of CHF patients

The mean Barthel index score of recruited CHF patients was 82.24 ± 21.26 points, suggesting their good ADL. However, only 2.5% of CHF patients were engaged in CR programs. In addition, 77.9% of them participated in daily activities/exercises, the top three of which were walking (73.8%), housework (27.9%) and jogging (21.3%). CHF patients were less involved in other low‐risk activities/exercises, like dancing (7.0%) and Tai Chi (1.6%). Despite strong evidence supporting the preventive advantages of CR, previous research has indicated that CR is underutilized by clinicians and patients.^23^, ^24^ The low participation rate in CR can be ascribed to various factors, including patient‐related factors, the level of medical care, socioeconomic factors, and national context. Within the scope of this study, the patient factors encompass two aspects: namely, high kinesiophobia scores and a low socioeconomic status. Fear of movement is a significant barrier to CR attendance and management in HF patients, which has been confirmed in both qualitative and quantitative studies.^25^, ^26^ Moreover, Sina Kianoush et al. found that the patients with a higher Social Vulnerability Index exhibited a low utilization rate of CR, who is mainly characterized by poverty, unemployment and educational attainment below high school level in terms of social and economic status.^27^ The problem of patient's non‐adherence to CR programs is still a challenge especially during the COVID‐19 Outbreak. We analyzed the causation of this phenomenon. The COVID‐19 pandemic has resulted in alterations to cardiovascular risk factors, and a persistent escalation in stress levels and apprehension within the population, including a heightened fear of physical activity.^28^, ^29^ Meanwhile, as a result of government‐imposed restrictions aimed at mitigating the spread of the virus, there was a concomitant reduction in both physical and social activity.^30^ This led to increased cardiovascular risk, depression, stress, and insomnia.^29^, ^31^ The negative health habits that people developed during the pandemic could persist for a long time, which may have particularly significant consequences for older individuals and females.^32^ Consequently, it is an urgent and imperative issue for national and regional authorities to enact a series of prevention and control measures targeting Long‐Covid Syndromes. Numerous studies have proven that Cardiac telerehabilitation (CTR) can improve cardiovascular disease management.^33^ The strength lies in reduction of unnecessary hospital visits, so as to reduce the risk of cluster infections. Notably, health professionals delivering CTR should be focused on recommendation for nutrition, physical activity, medication adherence, psychological status, correct distancing, and symptom monitoring and symptom management.^33^

Kinesiophobia was detected in 170/244 (69.5%) CHF patients, suggesting that kinesiophobia was highly prevalent in CHF patients, who had limited or low‐intensity daily activities/exercises. Previous studies reported higher Kinesiophobia scores (40.66 ± 5.56‐points) in patients with acute cardiovascular disease and elderly people with CHF than those in our findings, which may be attributed to the acute phase of disease, stronger fear on pain and negative emotions in the elderly.^13^, ^34^, ^35^ A growing number of studies have validated the high risk of hospitalization and mortality in HF patients with poor physical abilities.^36^ The moderate‐intensity aerobic exercise is the most effective rehabilitation for HF patients. Accumulating evidence have supported the benefits of high‐intensity interval exercise in improving physical capacity, alleviating clinical symptoms and enhancing the quality of life in HF patients.^37^ Individualized CR programs involving multiple types of daily activities/exercises within safe exercise loads are recommended to adult CHF patients. Approaches targeting kinesiophobia should also be introduced to increase the efficiency of CR programs.

### 4.3 Correlations among the cardiac function, health status, ADL, and kinesiophobia of CHF patients

Pairwise correlations were identified among the cardiac function, health status, ADL and kinesiophobia in CHF patients. CHF patients with the severer classification of cardiac function possessed worse ADL, health status and kinesiophobia. Cardiac function could mainly reflect the clinical manifestations, health status and prognosis of HF patients. Decreased exercise capacity is a main symptom of HF, manifested as fatigue, dyspnea, precordial pain, and intolerance to high‐intensity exercise.^38^ Nonetheless, the subjects in any classification of HF are recommended to receive exercise training.^6^ It is recommended that the promotion of participation in CR exercise among patients with worse cardiac function remains imperative. Additionally, the consideration of personalized comprehensive exercise regimens, such as active combined passive exercise and high‐intensity interval training, is advised to cope with the deterioration of cardiac function.

The presence of kinesiophobia poses a significant risk to the life and health of patients in numerous other diseases, associated with disability, depression, pain and even disuse.^39^, ^40^ Meanwhile, Kinesiophobia is also a substantial obstacle to CR, which is a common phenomenon in individuals with cardiovascular disease, stemming from the fear of experiencing recurring symptoms. Previous studies have also concluded that Patients in NYHA IV class exhibited higher TSK values than those in lower classes (*p* < .001).^41^ Our study revealed a positive association between heart function and Kinesiophobia score; however, heart function did not exert a significant influence on Kinesiophobia. This may be attributed to the inclusion of 148 individuals (60.7%) classified as NYHA Ⅱ in our study, while only a small proportion of patients with NYHA Ⅰ (6.6%, *n* = 16) and NYHA Ⅳ (5.7%, *n* = 14) class were included in this study. Current studies mainly highlight the correlation between kinesiophobia and activity status in patients with cardiovascular diseases, and influencing factors. However, there is currently a limited number of interventions available to address kinesiophobia. Sahin et al.^42^ demonstrated that exercise‐based CR programs significantly reduced the incidence of kinesiophobia in patients with coronary heart disease. These findings are notable as they contribute to a deeper comprehension of the complex relationship between exercise and fear of exercise. They clearly point out the need for a more individually tailored approach to meet the unique requirements of CHF patients with. kinesiophobia. At present, the therapeutic strategies of HF not only aim to reduce the mortality, but also focus on the improvement of the quality of life and health status. Comprehensive CR programs based on the cardiac function, ADL and kinesiophobia of HF patients are needed to reduce the medical cost and enhance the well‐being of affected people.

### 4.4 Mediational effects of kinesiophobia plus ADL on the cardiac function and health status of CHF patients

Our findings revealed that the cardiac function not only directly influenced the health status of CHF patients, but also indirectly influenced it through kinesiophobia and ADL. The cardiac function of HF patients is closely linked with the pulmonary, renal, endocrine and metabolic functions, often accompanied by pain, depression, gastrointestinal discomfort, fatigue and other complications.^43^ In clinical practice, cardiac function indicators and relevant symptoms, age‐related changes and comorbidities should be well concerned in HF patients. Maintaining an acceptable cardiac function is favorable to alleviate clinical symptoms, improve the exercise tolerance and keep a good health status.

Kinesiophobia and ADL mediated the influence of cardiac function on the health status of CHF patients, and their indirect effects accounted for 43.48% of the total effect. Notably, no significant difference in the mediational effect was detected between kinesiophobia and ADL, both being able to predict the health status. Somatic symptoms can be aggravated by negative emotions. As a negative psychological state, kinesiophobia leads to negative cognitive and emotional changes in CHF patients, and decreased ADL is an independent risk factor for acute HF onset and death.^10^, ^44^ Our findings highlighted the importance of early recognition of kinesiophobia and decreased ADL to improve the health status of CHF patients. Establishing a positive attitude through CR programs is essential to reduce the incidence, disability and mortality of HF.

## 5 CONCLUSION

CHF patients exhibit insufficient enthusiasm in CR programs, limited and low‐intensity daily activities/exercises, and prevalent kinesiophobia. Pairwise correlations exist among cardiac function, health status, ADL and kinesiophobia of CHF patients. The cardiac function not only directly impairs health status, but also indirectly through kinesiophobia and ADL, the mediational effects of which are equal. Kinesiophobia should be relieved and ADL enhanced to improve the health status and prolong the life expectancy of HF patients.

## AUTHOR CONTRIBUTIONS

Tang Yifan: Writing original draft, Investigation, Methodology, Formal analysis. Huang Yanling: Formal analysis, Investigation, Methodology. Wang Weiyun: Investigation, Data curation. Hu Xiaolin: Project administration, Supervision. Gu Zejuan: Project administration. Wang Rong: Project administration. Gao Chunhong: Writing—review and editing, Project administration, Conceptualization, Supervision.

## CONFLICT OF INTEREST STATEMENT

The authors declare no conflict of interest.

## Data Availability

The data that support the findings of this study are available on request from the corresponding author. The data are not publicly available due to privacy or ethical restrictions.
